# Hints on Some Points in Eye Diseases Occurring in Children

**Published:** 1887-10

**Authors:** Edgar A. Browne


### SELECTIONS.

### Hints on Some Points in Eye Diseases Occurring in Children

BY EDGAR A. BROWNE, M.D.

After discussing the subject somewhat at length, in its various aspects, he says: "Let us pass to a general cause, temporary in duration, giving rise to a localized ocular defect permanent in character. As a rule, fits occurring during the first dentition are not regarded as having any permanent effects. It is found, however, that a certain proportion of children who suffer from teething convulsions have zonular cataracts associated with a peculiar malformation of the permanent teeth about to be described. These cataracts often escape detection altogether, as the opacities are sometimes very slight, and only impair vision sufficiently to cause the child to be considered shortsighted. He sees more or less badly at a distance and holds his book close when reading, but he is able to rub on at school, and he may perhaps never be brought under the notice of a competent observer. Other cases, of course, are more pronounced, and the cataracts are dealt with by operation. The most curious association of this particular lenticular defect is with the permanent teeth. When zonular cataract is found with a history of infantile convulsions, we may expect to find the permanent teeth malformed; when zonular cataract is found without a history of fits, the teeth are likely to be well formed. The distinctive peculiarity of these teeth is the imperfection of the enamel. The dentine projects beyond the enamel edge like an overgrown schoolboy's legs through his trousers. Sometimes the enamel does not extend over more than a fourth of the surface of the tooth, and the dentine projects like a flange or spikelet. At other times the enamel covers the whole tooth, but toward the lingual edge there will be seen an irregular horizontal groove, or a number of small holes like the worm holes in antique furniture. Not only well-marked convulsions, but a single slight fit or attack of crowing inspiration will be followed by this defect in the enamel. As the surface of the tooth is divided by a more or less well-marked line, above which the enamel may possibly be smooth, but below which the dentine or imperfect enamel exhibits a grooved, rough, and honeycombed surface, having a rude resemblance to rocks fretted to a certain height by the intermittent action of the ocean tides, I have employed the term "tidal-mark tooth" in my teaching. The name is found to be sufficiently descriptive. It establishes the distinction from the notched and pegged tooth of congenital syphilis that is associated with keratitis and irido-choroiditis. Of course, the characteristics of both species of defects may be found united in the same individual, as the victims of congenital syphilis sometimes have fits and zonular cataracts. Occasionally the dentine projection from a tidal-marked tooth is broken off, leaving an apparently short tooth, with a concave border. This is liable to be mistaken for a syphilitic notch. But the tidal-mark tooth is not a pegged tooth within the limits of the extension of its enamel, whereas, the syphilitic tooth is pegged the whole extent of its corona and its enamel is complete.

Teeth in town dwellers are so liable to be misshapen that some little care is needful in deciding on the typical form that is due to a particular determining cause. An opportunity occasionally presents itself of studying the effect of the modifying cause uninfluenced by accidental circumstances, as when syphilis is engrafted on to an otherwise healthy stock, as in the following instance: A very healthy married couple began by having two healthy children who reached adult age perfect in every respect, the teeth being in every way (like the parents') admirably regular and well formed. While on a voyage the husband contracted syphilis. The elder children have well-formed teeth; those born after the accident have choroiditis, and the teeth are pegged and notched. The stock being originally healthy, the alteration in the form of the teeth may be taken to represent the effect of the specific poison unaffected by any other constitutional defect. The enamel was perfect. The relations between fits, tidal-mark teeth, and zonular cataracts is curious and interesting. My experience seems to teach me that if zonular cataracts are found with a history of fits, the tidal-mark teeth will certainly be developed; zonular cataracts without a history of fits are accompanied by well formed teeth, and in many cases of infantile convulsions the subsequent injury to the teeth is seen, although there is no obvious injury to the lens. It has been surmised that this remarkable injury to the enamel is due to stomatitis caused by mercury administered for the sake of curing fits. Putting aside the fact that the routine practitioner of the day is more likely to give bromide of potassium than gray powder in any form of fits, I have satisfied myself in certain cases that no mercurial has been given. Dentition is a process of accelerated local growth, in its essential character lying close to the narrow borderland that divides nutrition from inflammation. In a certain proportion of cases the borderland is over-stepped, and though the inflammation passes away with the eruption of the teeth, it is reasonable to suppose it leaves behind it a damaged enamel organ,* or perhaps it may be more correct to assume that the activity of the organ is lessened temporarily. Probably we need not look beyond aberrant nature for an explanation of the failure in the formation of the enamel.

*The grooves seen in the nails after exhausting illnesses have a somewhat similar significance. But the matrix of the nail is a permanent organ, and the growth of the nail is continuous, whereas the enamel organ is a temporary structure, and the tooth can be affected in its growth only during a very limited period.

The pathology of the lenticular change is even more obscure. It has been surmised that the mechanical jarring of the convulsions may have caused slight separation of the layers of the lens. But zonular cataract occurs in a certain number of cases without fits (and, as above stated, without tidal-mark teeth), and we must look somewhat deeper. Heredity seems to be not without influence. In one case under observation I have noted where the mother had fits, tidal-mark teeth, and zonular cataracts, her daughter had zonular cataracts only. A certain proportion of cases are certainly rickety. Whether this is more than an accidental circumstance, just as congenital syphilis is an accidental circumstance, I am not prepared to say. I have no means of judging whether rickety children are more prone than others of a perverted nutritional type, say of strumous stock or unhealthy surroundings, to teething convulsions. It may be they are; the intimate connection between the dental and ophthalmic divisions of the fifth in adult life is undoubted. All practical ophthalmic surgeons are acquainted with anomalous cases of amblyopia, asthenopia, and the elevation of intra-ocular tension due to dental irritation, and it is likely that a cause which may give rise to functional disturbance in adult tissues will inflict structural injury during the period of growth.

It has been already remarked that the lenticular opacity following teething convulsions is sometimes so slight as not to be suspected or even easily detected. There is a class of cases who suffer from asthenopia. The symptoms are pain in or above the eyes, neuralgia, headache, blepharitis, and vesicular conjunctivitis. Formerly, these cases would have been drugged with quinine, etc., etc., but nowadays every schoolboy knows that neuralgic headaches are due to faulty conditions of refraction, and spectacles are ordered. These cases, which I do not find described in books, differ from ordinary asthenopia, inasmuch as relief does not follow corrections of the refractive error. The vision may be very little below normal. On investigation, we find the history of one or more fits, tidal-mark teeth, and if we examine the lens with a plane mirror and a pretty high convex, we may find one or two minute tell-tale dots. A small proportion of cases of rebellious asthenopia will be found to have this history and this apparently insignificant injury to the lens. The correction of small amounts of astigmatism often fails to give any relief, as the pain proceeds not so much from the inequality in the curvature of the cornea as in the inequality of the index of refraction in the lens. Here, then, we have a far-reaching sequence from a cause very temporarily manifested. First, difficult eruption of teeth, fits, impairment of the temporary enamel organ, injury to the growing lens; then, as a permanent legacy, opacity of the lens, ranging in degree from complete cataract to a faint, scarcely perceptible opacity, amounting to little more than an annoyance in near work.—*Liverpool Medico-Chirurgical Journal.*

